# Effect of Intrauterine Smoke Exposure on microRNA-15a Expression in Human Lung Development and Subsequent Asthma Risk

**DOI:** 10.3390/healthcare8040536

**Published:** 2020-12-04

**Authors:** Sunita Sharma, Alvin T. Kho, Divya Chhabra, Kathleen Haley, Carrie Vyhlidal, Roger Gaedigk, J. Steven Leeder, Kelan G. Tantisira, Benjamin Raby, Scott T. Weiss

**Affiliations:** 1 Division of Pulmonary Sciences and Critical Care Medicine, University of Colorado School of Medicine, Aurora, CO 80045, USA; 2 Channing Division of Network Medicine, Brigham and Women’s Hospital, Boston, MA 02115, USA; alvin_kho@hms.harvard.edu (A.T.K.); rebar@channing.harvard.edu (B.R.); restw@channing.harvard.edu (S.T.W.); 3 Boston Children’s Hospital Informatics Program, Boston, MA 02115, USA; 4 National Jewish Health, Denver, CO 80206, USA; ChhabraD@NJHealth.org; 5 Division of Pulmonary and Critical Care, Department of Medicine, Brigham and Women’s Hospital, Boston, MA 02115, USA; khaley@rics.bwh.harvard.edu; 6 Children’s Mercy Hospital and Clinics, Kansas City, MO 64111, USA; cvyhlidal@cmh.edu (C.V.); rgaedigk@cmh.edu (R.G.); sleeder@cmh.edu (J.S.L.); 7 Division of Pediatric Pulmonary Medicine, University of California San Diego, San Diego, CA 92093, USA; rekgt@channing.harvard.edu; 8 Division of Pediatric Pulmonary Medicine, Children’s Hospital Boston, Boston, MA 02115, USA

**Keywords:** asthma, gene expression, in utero smoke, lung development, microRNAs

## Abstract

**Background:** In utero smoke (IUS) exposure is associated with asthma susceptibility. **Objective:** We sought to test the hypothesis that changes in miRNA expression by IUS exposure during human lung development is associated with asthma susceptibility. **Methods:** Gene expression was profiled from 53 IUS unexposed and 51 IUS exposed human fetal lung tissues. We tested for the differential expression of miRNAs across post-conception age and by IUS using linear models with covariate adjustment. We tested the IUS-associated miRNAs for association with their gene expression targets using pair-wise inverse correlation. Using our mouse model, we investigated the persistence of the IUS-associated miRNA signature using RT-PCR from the lungs of mouse pups with and without IUS at postnatal day 14. MiRNAs were then tested for association with asthma and exacerbations using whole blood gene expression profiles from Asthma BRIDGE. **Results:** Five miRNAs were differentially expressed across post-conception age (adjusted *p* < 0.0002) including two that were differentially expressed by IUS exposure in human fetal lung (*p* < 0.05). MiR-15a was differentially expressed by post-conception age (*p* = 0.00002), IUS exposure in human fetal lung (*p* = 0.005), and in the post-natal mouse lung (*p* = 0.01). MiR-15a was also associated with the in utero expression of *GSDMB* (adjusted *p* = 0.0002), a known childhood asthma gene and with asthma exacerbations (*p* = 0.0009) in Asthma BRIDGE. Thus, miR-15a is expressed during human lung development, is impacted by IUS exposure, regulates the intrauterine expression of asthma genes, and is associated with asthma severity. **Conclusions:** These results provide evidence for the role of miR-15a in the fetal origin of asthma.

## 1. Introduction

Asthma is characterized by airway inflammation, airway hyper-responsiveness, and reversible airflow obstruction. It is the most common chronic respiratory disease of childhood affecting 22 million people in the United States (U.S.), and is the most common cause of admissions to U.S. pediatric hospitals, and results in the highest number of missed school and work days each year in the U.S. [1,2].

Both genetic factors and environmental exposures have been shown to be involved in the pathogenesis of asthma. Substantial epidemiologic data supports a role for in utero smoke (IUS) exposure in the development of reduced lung function [3] and increased asthma susceptibility [4,5]. Despite the longstanding recognition that in utero exposures impact subsequent asthma susceptibility, the molecular characterization of these processes has been largely overlooked.

The “fetal origins hypothesis” [6] proposes that intrauterine exposures result in long-term changes in development that contribute to disease susceptibility later in life [7,8]. Since reduced neonatal lung function has been associated with lower lung function in childhood, wheezing, and an earlier diagnosis of asthma [9,10], it is believed that there are developmental origins of the disease.

Human lung development starts at approximately 4 weeks of gestation and can be classified into four distinct histologic stages: (1) pseudoglandular (weeks 7–17), (2) canalicular (weeks 17–26), (3) saccular (weeks 27–36), and (4) the alveolar (weeks 36–2 years) stages [11]. We previously investigated the gene expression profile of the normal human lung development [12] and demonstrated that there are critical molecular transitions that occur during lung development that are independent of these traditional histologic stages. Furthermore, we identified that genes in the Wnt-signaling pathway are differentially expressed between the pseudoglandular and canalicular stages of development and that these genes harbor genetic variants that are significantly associated with abnormal lung function in two childhood asthma cohorts [13]. These results demonstrate the ability of genomic profiling to identify novel biology and also suggest that the genomic profiling of early human fetal lung development may help in the identification of genes implicated in the pathogenesis of asthma.

MicroRNAs (miRNAs) are small non-coding RNA molecules that modulate physiological and pathological processes by regulating gene expression at the post-transcriptional level by repressing translation or decreasing mRNA stability [14]. Animal models demonstrate that miRNAs are an integral regulatory mechanism necessary to fine-tune the complex genomic profile of normal development [15,16,17,18]. MiRNAs have been associated with asthma and have been demonstrated to show promise as a possible biomarker for asthma susceptibility. Furthermore, tobacco smoke exposure has been previously shown to affect miRNA expression in the bronchial epithelium, with 28 miRNAs reported to be differentially expressed in current smokers compared to lifelong non-smokers and thus may impact disease susceptibility in these individuals [19]. Based on these observations, we hypothesized that the miRNA expression profile of human lung development is altered by IUS exposure, the effect of IUS exposure on the miRNA signature persists into the post-natal period, and impacts asthma susceptibility and disease severity later in life.

In our current work, we demonstrate the impact of IUS exposure on the miRNA signature of early human lung development. We identify miRNAs that are differentially expressed by IUS exposure during human lung development and demonstrate that these miRNAs regulate the intrauterine expression of known asthma susceptibility genes. Furthermore, we demonstrate that these developmental miRNAs are also associated with asthma severity in a well characterized asthma cohort, supporting an important role for developmental miRNAs in the pathogenesis of asthma later in life. Some of the results of these studies have been previously reported in the form of an abstract [20].

## 2. Materials and Methods

### 2.1. Gene Expression Profiling of Human Fetal Lung Tissue Samples

Human fetal lung tissue samples were acquired through the tissue retrieval program sponsored by the National Institute of Child Health and Development, the University of Maryland Brain and Tissue Bank for Developmental Disorders (Baltimore, MD, USA), and the Center for Birth Defects Research (University of Washington, Seattle, WA, USA) [13]. Gene expression profiles were generated using the Affymetrix Human 1.0 ST Array. All human and animal studies were approved by the Brigham and Women’s Hospital Institutional Review Board.

RNA was extracted from fetal tissue whole lung homogenate and RNA sample quality was assessed using the Agilent 2100 Bioanalyzer (Agilent Technologies, Inc.,Santa Clara, CA, USA). Genome-wide gene expression profiles were generated using the Affymetrix Human 1.0 ST Array. The pre-processing of the gene expression data was performed including background correction and quantile normalization prior to analysis. The probe with the highest variance for each miRNA was used as the representation of each miRNA. In total, ~200 of the known miRNAs were interrogated on this Affymetrix platform.

### 2.2. In Utero Smoke Exposure Assessment

We utilized our validated method to identify fetal lung tissues that had IUS exposure by measuring cotinine, a nicotine metabolite and established biomarker of tobacco smoke exposure, from the placental tissue corresponding to each of the human fetal lung tissues [21]. IUS unexposed samples had no detectable cotinine in the placenta, while samples with cotinine values of ≥7.5 ng/g tissue were considered for inclusion as IUS exposed samples. We have previously shown that a cotinine value ≥ 7.5 ng/g tissue in the placenta has a 78.7% sensitivity and 100% specificity to classify samples as IUS exposed [21].

### 2.3. Sample Selection for Analysis

In order to address the unmeasured confounders that exist in complex developmental gene expression data, a well-defined subset of samples was chosen for these analyses. Samples were matched based on age, gender, and principal components analysis (PCA) was used for outlier removal. PCA allows for matching based on variation in gene expression determined with measured and unmeasured variables. Samples matched for post-conception age and gender were then carried forward for differential expression.

### 2.4. Differential Expression of miRNAs across Human Lung Development

Differential miRNA expression across post-conception age was assessed using linear models adjusted for sex using the limma package in Bioconductor [22]. In order to determine the sex-specific effects on microRNA expression, a stratified analysis was performed by analyzing male and female subjects separately. Multiple comparisons adjustment was made using a Bonferonni correction. Additional details available in the Supplementary Materials.

### 2.5. Differential Gene Expression between IUS Unexposed and IUS Exposed Human Fetal Lung Tissue Samples

The developmentally regulated miRNAs were then tested for differential expression by IUS exposure using linear models adjusted for post-conception age and sex in limma [22]. A stratified analysis was performed by analyzing male and female subjects separately.

In order to determine whether developmentally regulated miRNAs that are differentially expressed by IUS exposure influence the intrauterine expression of known asthma genes, we next identified the gene expression (mRNA) targets of the IUS-associated miRNAs by comparing the inverse expression of the predicted mRNA target and the miRNAs in mirdb (http://mirdb.org/miRDB/) using the genome-wide gene expression profiles for these samples. Pathway enrichment analysis of the gene expression targets was performed in DAVID [23].

### 2.6. Investigating the Persistence of the miRNA Expression Signature of IUS into the Postnatal Period Using a Mouse Model of IUS Exposure

We used our mouse model [21,24] to investigate the effect of IUS exposure on miRNA expression in the early postnatal period. The “Principles of Laboratory Animal Care” formulated by the National Society for Medical Research were followed for the use of our mouse model. At postnatal day 14 (PN14), 4 IUS-exposed mouse pups and 4 IUS-unexposed mouse pups were sacrificed and their lungs harvested [25]. RT-PCR was performed for each of the miRNAs that were identified to be differentially expressed by IUS exposure in our human fetal lung analysis. The differential expression of these miRNAs by IUS exposure was performed using t-tests. In order to validate the impact of miRNA expression on asthma susceptibility gene expression, we used publicly available developmental mouse gene expression data to determine the correlations between miRNAs and the asthma susceptibility loci in the chromosome 17q locus (GSE21052).

### 2.7. MicroRNA Validation

We performed miRNA validation on a subset of 30 human fetal lung tissue samples using TaqMan (Life Technologies, Grand Island, NY, USA).

### 2.8. Association of the Human Fetal Lung miRNA Gene Expression Signature with Asthma Susceptibility and Disease Severity in Asthma BRIDGE

Asthma BRIDGE is a multicenter-collaborative effort to develop translational genomic datasets for asthma in North America [26]. Subjects provided informed consent prior to admission into their respective studies and the studies were approved by the Institutional Review Board of the Brigham and Women’s Hospital. Gene-expression profiles (Illumina Human HT-12 v4 array) were generated from whole blood in asthmatics and control subjects.

### 2.9. Differential Gene Expression between IUS Unexposed and IUS Exposed Subjects in the Asthma BRIDGE Cohort

Differential miRNA expression analysis between IUS exposure status was assessed using linear models adjusted for age, sex, and race in limma [22]. MiRNAs that were significantly differentially expressed by IUS exposure in both human fetal lung tissue and in whole blood from Asthma BRIDGE were then tested for association with asthma susceptibility and exacerbation by testing for differential expression between asthmatic subjects and non-asthmatic controls and between asthmatics with and without asthma exacerbations.

## 3. Results

### 3.1. Differential Expression of microRNAs by Post-Conception Age in Human Lung Development

In total, 104 samples that were matched based on post-conception age and sex were included in the human fetal lung gene expression analysis of IUS exposure. The baseline characteristics of these samples are shown in Table 1. Human fetal lung tissue samples were from the pseudoglandular stage of early lung development (post-conception age of 75–103 days). Samples that had an undetectable placental cotinine value were included as IUS unexposed samples (*n* = 53). Fifty-one samples that had placental cotinine values of ≥ 7.5 ng/g tissue represented the IUS exposed fetal lung tissue samples (mean placental cotinine = 59.4 ng/g tissue). There was a predominance of male subjects in both the IUS-unexposed and IUS-exposed samples.

Of the miRNAs that can be interrogated on the Affymetrix Human ST 1.0 array, a total of 162 were expressed in human fetal lung tissue. Of these 162 miRNAs, differential expression of miRNAs across human lung development was performed using linear models adjusted for sex in the IUS unexposed samples only. We identified five miRNAs that are differentially expressed at an adjusted *p* < 0.05 by post-conception age during human lung development (Table 2). Of note, miR-330 was the most differentially expressed by post-conception age (adjusted *p* = 0.009) during human fetal lung development. A stratified analysis between male and female samples was performed to identify sex-specific differences in miRNA expression across human lung development. Interestingly, three miRNAs were identified to be differentially expressed in male subjects, but not in female subjects, (Table 3) demonstrating sex-specific differences in miRNAs during early human fetal lung development.

### 3.2. Differential miRNA Expression between IUS-Unexposed and IUS-Exposed Human Fetal Lung Tissue

Differential miRNA expression analysis between IUS-unexposed and IUS-exposed human fetal lung tissue samples using linear models adjusted for post-conception age and sex identified two miRNAs that were differentially expressed at an adjusted *p* < 0.05, including miR-155 (*p* = 0.0009) and miR-15a (*p* = 0.003) (Table 4).

The gene targets of the two developmental miRNAs that were differentially expressed by IUS exposure during human fetal lung development include genes that have been previously associated with asthma susceptibility in published genome-wide association studies (GWAS) of asthma, including an association of miR-15a with Gasdermin B (*GSDMB*) [27] and miR-155 with *thymic stromal lymphopoietin* (*TSLP*) [28] (Table 4).

### 3.3. Persistence of the miRNA Expression Signature of IUS in the Postnatal Period Using a Mouse Model of IUS Exposure

**Figure 1:**
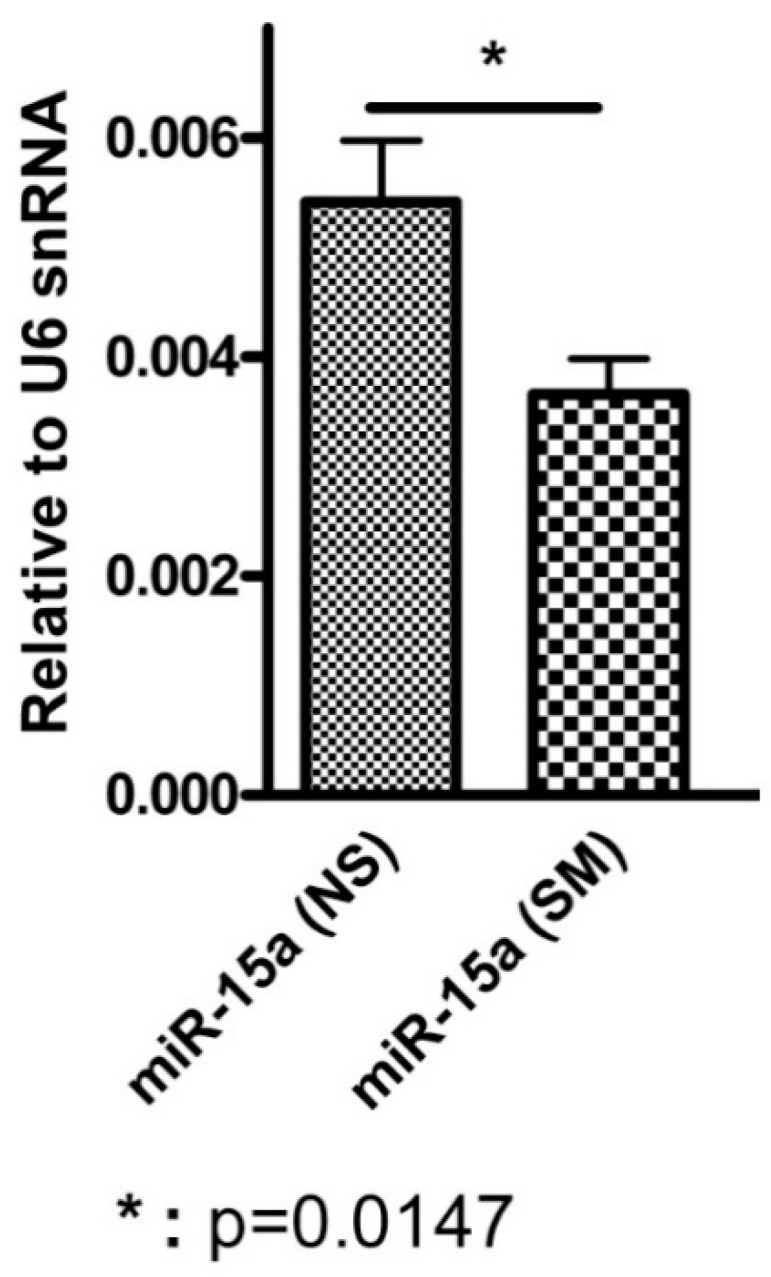
Differential expression of miR-15a by IUS exposure in postnatal mouse lung. NS are mice without intrauterine smoke exposure and SM are mice exposed to intrauterine smoke.

Using our mouse model of IUS exposure, we investigated the persistence of the IUS signature of miRNA expression into the postnatal period by performing the RT-PCR of miR-155 and miR-15a in the lungs of mouse pups with (*n* = 4) and without IUS (*n* = 4) exposure at postnatal day 14 (PN14). Although miR-155 was not significantly differentially expressed by IUS exposure in the postnatal period in our mouse model, we did confirm that IUS exposure results in a decreased miR-15a expression into the alveolar stage of development. We demonstrated that miR-15a is significantly differentially expressed between IUS-exposed and IUS-unexposed mouse lung at PN14 (*p* = 0.01, Figure 1). Using publicly available mouse developmental gene expression data (GSE21052) we also confirmed the correlation of miR-15a miR-15b with the asthma susceptibility chromosome 17q locus including GSDMA (R = −0.56923) and ORMDL3 (R = +0.6044).

Thus, miR-15a is differentially expressed by IUS exposure during early human lung development, and this pattern persists into the postnatal period in our mouse model.

### 3.4. Association of miR-15a Expression with IUS Exposure and Asthma Susceptibility and Disease Severity in Asthma BRIDGE

In order to investigate whether the human fetal lung miR-15a expression signature of IUS exposure influences asthma susceptibility, we then tested for the differential expression of miR-15a with a history of IUS exposure, and the association of this miR-15a with asthma susceptibility and disease severity using whole blood gene expression profiles from the Asthma BRIDGE cohort. The baseline characteristics of the Asthma BRIDGE biorepository subjects used in these analyses are shown in Table 5. Since the most robust evidence for differential expression resulting from IUS exposure in the human fetal lung was found for miR-15a, our analyses in Asthma BRIDGE were performed for miR-15a only.

Similar to the human fetal lung analysis, we found that miR-15a was differentially expressed by a history of IUS exposure in Asthma BRIDGE with higher levels of expression noted in the nonsmokers (*p* = 0.03, Table 6). Although miR-15a was not noted to be differentially expressed between asthmatics and non-asthmatic controls (*p* = 0.56) in asthma BRIDGE, miR-15a expression was noted to be associated with markers of asthma severity. Of note, miR-15a was significantly differentially expressed between asthmatics with and without asthma exacerbations (Table 6). Notably, mir15a was only marginally associated with log10 eosinophil level in this population (*p* = 0.052). Thus, additional investigations are necessary to determine the impact of microRNA expression on known asthma endotypes.

## 4. Discussion

By applying genomic technologies to the investigation of human lung development, we demonstrated that miR-15a is both differentially expressed by post-conception age and is impacted by IUS exposure during early human lung development. Furthermore, this signature persists into the postnatal period, independently of additional postnatal environmental tobacco smoke exposure. IUS-associated miRNAs also regulate the intrauterine expression of a known asthma susceptibility gene and are associated with asthma severity later in life. These results provide proof of concept that the miRNA signature that results from IUS exposure persists into the later stages of development, and that this epigenomic signature may be implicated in asthma pathogenesis later in life. Furthermore, these results provide evidence for the role of miRNAs in the fetal origins of asthma and begins to elucidate the largely unknown biologic mechanisms of the developmental origins of asthma for which there is supportive epidemiologic data, including the influence of IUS exposure on asthma susceptibility.

MicroRNA expression patterns are highly conserved between human fetal and adult tissues [29], and are known to fine-tune the relative expression of multiple target genes [30,31]. MicroRNAs are considered essential to the developmental process but are yet to be investigated in the developmental origin of asthma. Thus, we characterized the miRNA expression profile of normal human lung development, identified how this signature is impacted by IUS exposure, and demonstrated that these developmental IUS-associated miRNAs are associated with asthma severity. Furthermore, we demonstrated that these patterns are preserved in both human lung tissue and peripheral blood samples, suggesting that miR-15a expression may have potential as a biomarker of IUS exposure.

Previous mouse models of asthma identified 21 miRNAs that were differentially expressed between mice with allergic airway inflammation and controls including several of the miRNAs that we identified to be differentially expressed in human lung development [32]. In addition, a mouse model of house dust-mite-induced allergic asthma demonstrated that the levels of several developmental miRNAs were elevated in comparison to the controls [33].

We showed that developmental IUS-associated miRNAs (both miR-155 and miR-15a) regulate the intrauterine expression of known asthma susceptibility genes during human lung development. These results are consistent with recent studies that have shown that genetic variants associated with disease susceptibility in GWAS are more likely to regulate gene expression, and to be developmental genes [34]. The gene targets of both miR-155 and miR-15a include genes that have been previously associated with asthma susceptibility in published asthma GWAS including *GSDMB* [27] and *TSLP* [28]. Notably, miR-15a was highly associated with the regulation of the *GSDMB* gene, which resides on the chromosome 17q locus that has become the most replicated childhood asthma association in previously published GWAS. These results not only demonstrate the importance of miRNAs in human lung development, but also support a plausible developmental role for miR-15a in the pathogenesis of asthma.

Tobacco smoke exposure has been previously shown to effect miRNA expression in the bronchial epithelium, with 28 miRNAs reported to be differentially expressed in current smokers compared to lifelong non-smokers [19] including miR-15a, which was downregulated by smoke exposure. Rats exposed to cigarette smoke develop extensive alteration in the expression of miRNAs in the lung [19]. Extensive work has demonstrated that miRNAs are associated with cancer and the increased expression of numerous oncogenes. MiR-15a has been associated with lung cancer and lung cancer-specific survival [35,36]. It is an important regulator of multiple oncogenic proteins including bcl-2, ras, and c-myc proteins. Furthermore, miR-15a expression in the blood of patients with oral squamous cell cancer has been used as a biomarker for tumor staging [37] and demonstrates the promise of using blood miRNA expression as biomarkers of disease.

Although miR-15a has not been directly associated with asthma pathogenesis, a mouse model of graft versus host disease found that the over-expression of miR-15a in cord blood leads to the inhibition of the FOXP3 gene. Furthermore, miR-15a plays an important role in mediating the suppressive function of cord blood regulatory T cells (Tregs) [38]. Foxp3 is known to activate Tregs and suppress NKT cells in asthma [39]. Both interleukin-10 (IL-10) and transforming growth factor beta (TGFβ) modulate the expression of FoxP3 and influence Treg cell number and function [40]. Tregs play an essential role in early immune programming and are known to shape the immune response towards an allergic or tolerant state. Previous investigations have demonstrated that microbial sensitization results in lower Treg percentages and this profile resulted in postnatal atopy [41]. Interestingly, intrauterine exposure to cigarette smoke exposure was shown to reduce cytotoxic T-lymphocyte activity and also results in increased levels of thymic Treg cells in a time-dependent manner. These previous studies suggest that the children of smoking mothers may be less able to mount appropriate adaptive immune responses later in life [42]. Furthermore, these studies demonstrate a possible mechanism whereby both miR-15a expression and IUS exposure may influence asthma severity.

Previous studies of the role of miR-155 and miR-15a in human lung development, the role of IUS exposure on their expression patterns, and their association with the developmental origins of asthma are extremely limited. However, Rodriguez and colleagues have demonstrated that the knockout of miR-155 results in the development of an asthma-like phenotype including inflammatory cell infiltration into the airway and airway remodeling [43]. Furthermore, miR-155 has been shown to have a central role in the acquisition and maintenance of the Th2 phenotype by modulating the response of human macrophages to IL13 [44]. The inhibition of miR-155 has also been shown to lead to an increase in transcription factors involved in the generation of a Th2 microenvironment implicating this miRNA in the pathogenesis of asthma.

Notably, our findings suggest that the impact of IUS exposure on microRNA expression differs from the effect of post-conception age alone. This is congruent to previously published developmental data demonstrating that the impact of IUS exposure changes across the developmental timeframe and that the effects may increase at later stages of gestation. The investigation of later developmental stages may help to elucidate these effects.

Although we undertook a comprehensive analysis of the developmental signature of miRNAs and the influence of IUS exposure on this signature, several limitations of our analysis must be addressed. Although the Affymetrix array allows the interrogation of approximately 200 of the known miRNAs, this platform allows for only mature miRNA analysis and does not provide a more extensive investigation of novel and other known miRNAs. Furthermore, our analysis of human lung development is limited to fetal lung tissue samples from the early stages of development. Therefore, we were unable to investigate the miRNA profile during later human lung development. Since human fetal tissues from the later stages of gestation are difficult to obtain, we utilized our mouse model of IUS exposure to evaluate the later stages of development. Although we acknowledge that the investigation of miRNA expression patterns during the later stages of human lung development may allow us to gain further insights into the pathogenesis of respiratory diseases like asthma, the use of our mouse model allows for some additional insights regarding the miRNA expression changes that occur during the later stages of gestation and into the post-natal period. In addition, these results do suggest that the further investigation of these miRNAs as a biomarker of IUS exposure and asthma susceptibility and disease severity is warranted.

## 5. Conclusions

In this study, we identified developmental miRNAs that are differentially expressed during early human lung development, impacted by IUS exposure, and influence the intrauterine expression of known asthma genes. In addition, we demonstrate that developmentally relevant miRNAs influence asthma severity in asthmatic patients. Our results do not only provide evidence for a role for miR-15a in the fetal origins of asthma, but should also motivate future investigations on the role of miRNAs in the pathogenesis of other respiratory diseases in which IUS exposure has been implicated in disease susceptibility.

## Figures and Tables

**Table 1:** Baseline characteristics for the human fetal lung tissue samples utilized in these analyses.

Variable	Intrauterine Smoke Exposed (*n* = 51)	Intrauterine Smoke Unexposed (*n* = 53)
Post-conception age (days) *	27.96 (15.65)	27.64 (16)
Male sex (percentage)	26 (51%)	29 (54%)
Cotinine value (ng/g tissue)	59.44 (35.6)	0

* mean (standard deviation).

**Table 2:** Differential expression of microRNAs by post-conception age.

MicroRNA	*p* Value for Association with Post-Conception Age *
miR-330	0.0009
miR-155	0.002
miR-31	0.002
miR-15a	0.002
miR-455	0.003

* microRNA is differentially expressed by post-conception age in IUS-unexposed samples capturing only the impact of gestational age on microRNA expression.

**Table 3:** Sex-specific differential expression of microRNAs by post-conception age during human lung development.

MicroRNA	*p* Value for Association with Post-Conception Age *
miR-153-2	5.61 × 10^−6^
miR-196a	0.001
miR-184	0.002

* Differential expression of microRNA by the post-conception age in male subjects only.

**Table 4:** Association of developmental IUS-associated miRNAS with the intrauterine expression of known asthma genome-wide association studies (GWAS) genes.

MiRNA	Association with IUS Exposure (*p* < 0.05)	Asthma GWAS Gene (Association *p* Value)	Selected Pathway Enrichment Analysis of MiRNA Gene Expression Targets (*p* < 0.05)
miR-155	0.0009	*TSLP* (*p* = 2.2 × 10^−4^)	RNA splicing, in utero development
miR-15a	0.003	*GSDMB* (*p* = 3.3 × 10^−3^)	Regulation of transcription

**Table 5:** Characteristics of the Asthma BRIDGE subjects used to test for the association of miR-15a with asthma susceptibility and disease severity.

	Asthma (*n* = 865)	Non-Asthmatic Controls (*n* = 116)
Age years (mean, SD)	12.55 (6.8)	26.45 (1.7)
Male sex (%)		
Caucasian race (number, %)	457 (52%)	31 (27%)
IUS	90 (10%)	5 (4%)

**Table 6:** Association of miRNA with IUS and asthma severity in Asthma BRIDGE.

MiRNA	Cell Type Expression	Phenotype	Association *p* Value
miR-15a	Whole blood	IUS	0.03
		Asthma exacerbations	0.009

## Supplementary Materials

The following are available online at https://www.mdpi.com/2227-9032/8/4/536/s1.
