# Supplementary material for: Effect of Intrauterine Smoke Exposure on microRNA-15a Expression in Human Lung Development and Subsequent Asthma Risk
Source: Healthcare (Basel). 2020 Dec 4;8(4):536. doi: 10.3390/healthcare8040536 (PMC7761806; doi:10.3390/healthcare8040536)
Supplement: Supplementary file 1 [file healthcare-08-00536-s001.pdf]

Effect of intrauterine smoke exposure on microRNA-15a expression in human lung development and its association with asthma risk

Sunita Sharma[1*], Alvin T. Kho[2,3*], Divya Chhabra[4], Kathy J. Haley[5], Carrie A. Vyhlidal[6], Roger Gaedigk[6], J. Steven Leeder[6], Kelan G. Tantisira[7], Benjamin A. Raby[2,7,] and Scott T. Weiss[2]

Supplementary Materials

Materials and Methods

Gene expression profiling of human fetal lung tissue samples

Human Fetal Lung Gene Expression Profiling

Human fetal lung tissue samples were acquired through the tissue retrieval program sponsored by the National Institute of Child Health and Development, the University of Maryland Brain and Tissue Bank for Developmental Disorders (Baltimore, MD), and the Center for Birth Defects Research (University of Washington, Seattle, WA) as previously described (13). The collection of these tissues has been designated an institutional review board (IRB)-exempt protocol by the University of Missouri–Kansas City Pediatric IRB. RNA was isolated from human fetal lung tissue samples using the RNeasy mini kit (Qiagen, Valencia, CA)) according to protocol.

Sample quality was assessed using the Agilent 2100 Bioanalyzer (Santa Clara, CA). Genome-wide gene expression profiles were generated using the Affymetrix Human 1.0 ST Array. Pre-processing of the gene expression data was performed including background correction and quantile normalization prior to analysis. The probe with the highest variance for each miRNA was used as the representation of each miRNA. In total, ~200 of the known miRNAs are interrogated on this Affymetrix platform.

In Utero Smoke Exposure Assessment

We utilized our validated method to identify fetal lung tissues that had IUS exposure by measuring cotinine, a nicotine metabolite and established biomarker of tobacco smoke exposure, from the placental tissue corresponding to each of the human fetal lung tissue samples (21). Cotinine determination was made using direct Elisa assays using crude placental tissue extracts as described in detail (21). We have previously shown that a cotinine value ≥ 7.5 ng/g tissue in the placenta has a 78.7% sensitivity and 100% specificity to classify samples as IUS exposed (21). IUS unexposed samples had no detectable cotinine in the placenta, while samples with cotinine values of ≥ 7.5ng/g tissue were considered for inclusion as IUS exposed samples.

Sample selection for analysis

In order to address unmeasured confounders that exist in complex developmental gene expression data, a well-defined subset of samples was chosen for these analyses. Samples were matched based on age, gender, and using principal components analysis (PCA) for outlier removal. PCA allows for matching based on variation in gene expression determined with measured and unmeasured variables. Samples matched for post-conception age and gender were then carried forward for differential expression.

Differential expression of miRNAs across human lung development

Filtering was performed prior to differential expression analysis to remove miRNAs with either undetectable expression levels in fetal lung tissue or that demonstrated minimal expression variability across fetal lung tissue samples. Differential miRNA expression across post-

conception age during human lung development was assessed using linear models adjusted for sex using the *limma* package in Bioconductor(22). In addition, a stratified analysis was performed to evaluate for sex-specific differences in gene expression by analyzing male and female subjects separately. MiRNAs were found to be significantly differentially expressed if they met adjustment for multiple comparisons using a stringent Bonferonni correction and were carried forward for additional analyses.

Differential gene expression between IUS unexposed and IUS exposed human fetal lung tissue

The developmentally regulated miRNAs were then tested for differential expression between IUS exposed and IUS unexposed samples using linear models adjusted for post-conception age and gender in *limma* (22). In addition, a stratified analysis was performed to evaluate for sex-specific changes in gene expression due to IUS exposure by analyzing male and female subjects separately.

In order to determine whether developmentally regulated miRNAs that are differentially expressed by IUS exposure influence the intrauterine expression of known asthma genes, we next identified the gene expression (mRNA) targets of these developmentally regulated and IUS-associated miRNAs by comparing the inverse expression of the predicted mRNA target and the miRNAs in mirdb (http://mirdb.org/miRDB/) using the genome-wide gene expression profiles for these samples. The gene targets of 2 of the significantly developmental IUS-associated miRNAs were identified and then it was determined if any of the gene targets were known to be asthma susceptibility genes from published genome-wide association studies (GWAS) of asthma. Pathway enrichment analysis of the gene expression targets for these two miRNAs was then performed in DAVID(23).

Persistence of the miRNA expression signature of IUS in the postnatal period using a mouse model of IUS exposure

Since human fetal lung tissue samples from the late stages of gestation are difficult to obtain, we used our adapted mouse model of IUS exposure (21, 24) to investigate the persistence of the miRNA expression pattern of IUS exposure in the early postnatal period. We used a whole body cigarette smoke exposure model (Teague Enterprises, catalog number TE-10z). C57BL/6 females were acclimated to cigarette smoke reaching the goal of 2 hours/day for 6 days a week. The females were then mated to non-smoke exposed males. Total particulates were sampled daily, and kept between 100-200/m^3 throughout the pregnancy(25). In the postnatal period, 4 IUS-exposed mouse pups and 4 IUS unexposed mouse pups were sacrificed and their lungs harvested for miRNA expression analysis. RT-PCR was performed for each of the miRNAs that were identified to be differentially expressed by IUS exposure in our human fetal lung tissue analysis. Differential expression of these miRNAs by IUS exposure in the postnatal period was performed using t-tests for association.

MicroRNA validation

We performed miRNA validation in a subset of 30 human fetal lung tissue samples using TaqMan (Life Technologies, Grand Island, NY). MicroRNA validation was performed if the

Association of the human fetal lung miRNA gene expression signature with asthma susceptibility and disease severity in Asthma BRIDGE

Asthma BRIDGE Biorepository

Asthma BRIDGE is a multicenter-collaborative effort to develop well-characterized translational genomic datasets for asthma in North America(26). Samples were collected through October 2011 from among more than 14,000 subjects studied by the EVE Consortium, providing

a broad representation of the North American asthmatic population. Informed consent was obtained from each study participant before study enrollment. Genome-wide gene-expression profiles (Illumina Human HT-12 v4 array) were generated from whole blood in asthmatics and non-asthmatic control subjects. Pre-processing of the gene expression data was performed and quantile normalized prior to analysis. In addition, probes that did not map to known genes were removed from analysis. Extensive phenotypic information including a detailed questionnaire that includes medical information including history of IUS exposure is available for these subjects.

Differential gene expression between IUS unexposed and IUS exposed subjects in the Asthma BRIDGE cohort

Similar to the human fetal lung miRNA expression analysis, differential miRNA expression analysis between IUS exposed and IUS unexposed subjects was assessed using linear models adjusted for age, gender, and race in the *limma* package in Bioconductor(22). In order to assess the effects of intra-uterine nicotine exposure alone, current smokers and those with a history of active environmental tobacco exposure were removed from this analysis. A stratified analysis was performed to look for sex-specific miRNA expression changes due to IUS exposure. MiRNAs that were significantly differentially expressed by IUS exposure in both human fetal lung tissue and by IUS exposure in whole blood gene expression profiles from Asthma BRIDGE were then tested for association with asthma susceptibility and disease severity in the asthma BRIDGE cohort.

Association of human fetal lung IUS miRNA expression signature and asthma in the Asthma BRIDGE cohort

MicroRNAs that were differentially expressed by IUS exposure in human fetal lung tissue, differentially expressed by IUS exposure in the postnatal period in our mouse model, and

differentially expressed by IUS in Asthma BRIDGE, were then tested for association with asthma susceptibility and disease severity. MiRNAs were tested for differential expression between asthmatic subjects and non-asthmatic controls and between asthmatics with and without asthma exacerbations.
